# Prevalence and associated factors of common mental disorders among internally displaced people by armed conflict in Cabo Delgado, Mozambique: a cross-sectional community-based study

**DOI:** 10.3389/fpubh.2024.1371598

**Published:** 2024-04-16

**Authors:** Naisa Manafe, Hamida Ismael-Mulungo, Fábio Ponda, Palmira F. Dos Santos, Flávio Mandlate, Vasco F. J. Cumbe, Ana Olga Mocumbi, Maria R. Oliveira Martins

**Affiliations:** ^1^Instituto Nacional de Saúde, Maputo, Mozambique; ^2^Global Health and Tropical Medicine, Instituto de Higiene e Medicina Tropical, Universidade Nova de Lisboa, Lisbon, Portugal; ^3^Faculty of Medicine, Eduardo Mondlane University, Maputo, Mozambique; ^4^Mental Health Department, Ministry of Health, Provincial Health Directorate of Sofala, Beira, Mozambique

**Keywords:** post-traumatic stress disorder, anxiety, depression, internally displaced persons, humanitarian emergency, Cabo Delgado, Mozambique

## Abstract

**Background:**

Humanitarian emergencies are a major global health challenge with the potential to have a profound impact on people’s mental and psychological health. Displacement is a traumatic event that disrupts families and affects physical and psychological health at all ages. A person may endure or witness a traumatic incident, such as being exposed to war, and, as a result, develop post-traumatic stress disorder (PTSD). There is a lack of information about post-traumatic stress disorder, depression, and anxiety disorder in low and middle-income countries in humanitarian emergency contexts such as Mozambique. This study aimed to assess the prevalence of PTSD, depression, and anxiety, and associated factors among armed conflict survivors in Cabo Delgado, north region of Mozambique in 2023.

**Methods:**

A community-based cross-sectional study was conducted between January and April 2023 among 750 participants, who were selected by convenience. A face-to-face interview used the Primary Care Post-Traumatic Stress Disorder Checklist (PC-PTSD-5) to evaluate PTSD, the Generalized Anxiety Disorder Scale (GAD-7) to evaluate anxiety and the Patient Health Questionnaire – Mozambique (PHQ-9 MZ) to evaluate depression. The association between PTSD and demographic and psychosocial characteristics was analyzed using bivariate and multivariable binary logistic regression. We used a 5% significance level.

**Results:**

The three mental disorders assessed were highly prevalent in our sample with 74.3% PTSD, 63.8% depression, and 40.0% anxiety. The chance of developing PTSD was higher in females (AOR = 2.30, 95% CI 1.50–3.51), in patients with depression symptoms (AOR = 8.27, 95% CI = 4.97–13.74) and anxiety symptoms (AOR = 1.45, 95% CI = 0.84–2.50).

**Conclusion:**

This study reported that the prevalence of PTSD, depression, and anxiety were high. Patients having depressive symptoms, anxiety symptoms, and being female are more at risk of developing PTSD. There is a need to integrate screening for common mental disorders in the context of humanitarian emergencies and its adapted integration of psychosocial interventions.

## Introduction

Displacement due to armed conflict in developing countries is increasing (1, 2). Displacement is a traumatic event that disrupts families and affects physical and psychological health at all ages (3–5). At least two-thirds of the countries in Africa have experienced conflicts leading to the displacement of millions of people (6).

Epidemiological evidence shows that the burden of mental disorders is becoming higher, particularly in post-conflict and conflict-affected populations (7, 8).

Mental disorders are a significant public health problem and 14% of the total burden of disease has been attributed to neuropsychiatric disorders including depression and other common mental disorders (9). Additionally, the proportion of global Disability Adjusted Life Years (DALYs) attributed to mental disorders increased to 4.9% (3.9–6.1) and Years lived with disability (YLDs) contributed to most of the mental disorder burden, with 125.3 million YLDs (95% UI 93.0–163.2; 14.6% [12.2–16.8] of global YLDs) in 2019 (10). Exposure to repeated trauma and extreme violence such as torture is associated with an increased risk for a mental disorder, including post-traumatic stress disorder, depression, anxiety, schizophrenia, and bipolar disorder (7, 11–13).

Recent meta-analyses of several studies published between1980 and 2017, where estimated the prevalence of PTSD (96/129; 74.4% of the studies), depression (70/129; 54.3% of the studies), and anxiety (38/129; 29.4% of the studies) identified a relationship between exposure to different types of disaster and conflict-related events and mental health disorders including anxiety, depression, and PTSD (7). The prevalence of mental health diseases was 21% (95% CI 18.8–25.7) at the point in time in the conflict-affected populations assessed (7).

According to the results of another meta-analysis, an estimated 242 million adult war survivors living in post-conflict areas were affected by PTSD, while major depressive disorder affected an estimated 238 million and 117 million suffering from both conditions (14). According to the findings of this meta-analysis, the estimated adult war survivors with PTSD, major depression, and both conditions in Mozambique were 2,339,450 (95% CI, 1,919,902–2,785,528), 2,296,218 (95% CI, 1,933,657–2,679,412) and 1,124,917 (95% CI, 695,524–1,557,525), respectively. (14).

According to studies of the general population, PTSD prevalence ranges between 1 and 5% (15, 16), while it has been shown to range from 3 to 58% in high-risk groups, such as those in conflict areas (16). Another systematic review reported that 3 to 88% of people in the general population have PTSD (7, 17). This is the case in Nepal and Palestine where PTSD prevalence is 53.4% (18) and 68.9% (19) respectively. Additionally, cross-sectional studies conducted among Internally Displaced People (IDP) assessment at community revealed that the prevalence of PTSD was 63% in Nigeria (20), 19.3% in Morocco (21), 28% in South Sudan (22), and 58.4% in the north (23) of Ethiopia.

On the other hand, mental health affects the immune system, damaging the body’s immunity and defenses and leaving the individual more susceptible to infections; the endocrine system, increasing or decreasing the production of certain hormones; the nervous system, interfering with the production of neurotoxins (related to diseases such as Parkinson’s and Alzheimer’s disease), among other problems (24).

Mozambique hosts nearly 32,000 refugees and asylum-seekers, while more than one million people remain displaced internally due to violence perpetrated by non-state armed groups and the devastating impact of the climate crisis (Tropical Cyclone Gombe in March 2022) – where Mozambique is one of the most adversely affected countries in the world (25).

In October 2017, violence erupted in Cabo Delgado, northern Mozambique, when armed men occupied the Mocimboa da Praia district. This violence perpetrated by non-state armed groups worsened in 2020 resulting in an unprecedented humanitarian crisis with close to 1 million people living in a situation of protracted displacement (26). Violence against civilians continued such as killing, beating, extortion, widespread damage to property and core public services, severe violations of children’s rights, and conflict-related sexual violence (27). Due to this violence, 2000 civilians died and around 34% of people and 28% of families including 353,601 children, were displaced/forced to move to different locations within Cabo Delgado province and other regions of Mozambique such as Sofala and Zambezia in the center and Nampula and Niassa in the north (28). The main destinations of IDP arrivals in Cabo Delgado were Pemba, Metuge, Mueda, Ancuabe, and Montepuez districts (29, 30), where displaced people were initially housed in transitional accommodation centers and later resettled or returned to their places of origin. The IDPs still live in small, overcrowded temporary shelters in the camps, without sufficient food, clean water, or toilets. Their lives are on hold, and their futures are uncertain (26–29). Individuals with an experience of abuse/violence were at risk of increasing mental health problems.

Screening is effective only when combined with high-quality services for mental well-being.

One of the challenges to ensuring appropriate services for IDP in Cabo Delgado is the lack of statistical data on the group’s mental health status.

Despite the high prevalence of PTSD, depression, and anxiety in conflict areas around the world, data on the prevalence of PTSD, depression, and anxiety among IDPs in Mozambique, where we lived in a scenario of armed conflict (during 16 years of civil war), current terrorist attacks and violence since 2017, and recurring natural disasters are scarce. It is imperative to conduct research in this field to provide scientific support for the formulation of prevention and treatment plans for mental health issues during current and future civilian attacks or natural disasters. This study aims to assess the prevalence of symptoms and associated factors of post-traumatic stress disorder, depression, and anxiety among those who have experienced traumatic events during the armed conflict in Cabo Delgado province.

## Materials and methods

### Study design, period, and settings

A community-based, cross-sectional study was conducted with Internally Displaced Persons aged 14 years and over between January 2023 and April 2023.

Internally displaced persons (IDPs), according to the United Nations Guiding Principles on Internal Displacement, are “persons or groups of persons who have been forced or obliged to flee or to leave their homes or places of habitual residence, in particular as a result of or to avoid the effects of armed conflict, situations of generalized violence, violations of human rights or natural or human-made disasters, and who have not crossed an internationally recognized state border (31). In this study, an IDP was considered as someone who answered the questionnaire-screening questions that they had been forced to flee their homes because of the armed conflict and currently living in the IDP resettlement center.

Participants were excluded in cases of mental disability at the time of the survey that impeded their ability to competently consent and people who did not want to talk about their traumatic experience.

The present study was conducted in the *25 de Junho* resettlement center in Metuge district, Cabo Delgado Province, north of Mozambique. Cabo Delgado’s capital is the city of Pemba, located about 2,600 km north of Maputo, the country’s capital. The province has an area of 82,625 km^2^ and had, in 2017, a population of 2,333,278 inhabitants. The province of Cabo Delgado is divided into 17 districts and has 5 municipalities: Chiure, Mocimboa da Praia, Montepuez, Mueda, and Pemba (32).

Metuge is the closest city to Pemba (34 km), with 5 primary healthcare serving a population of 91,000 and 119,317 IDPs resettled (30).

**Figure 1:**
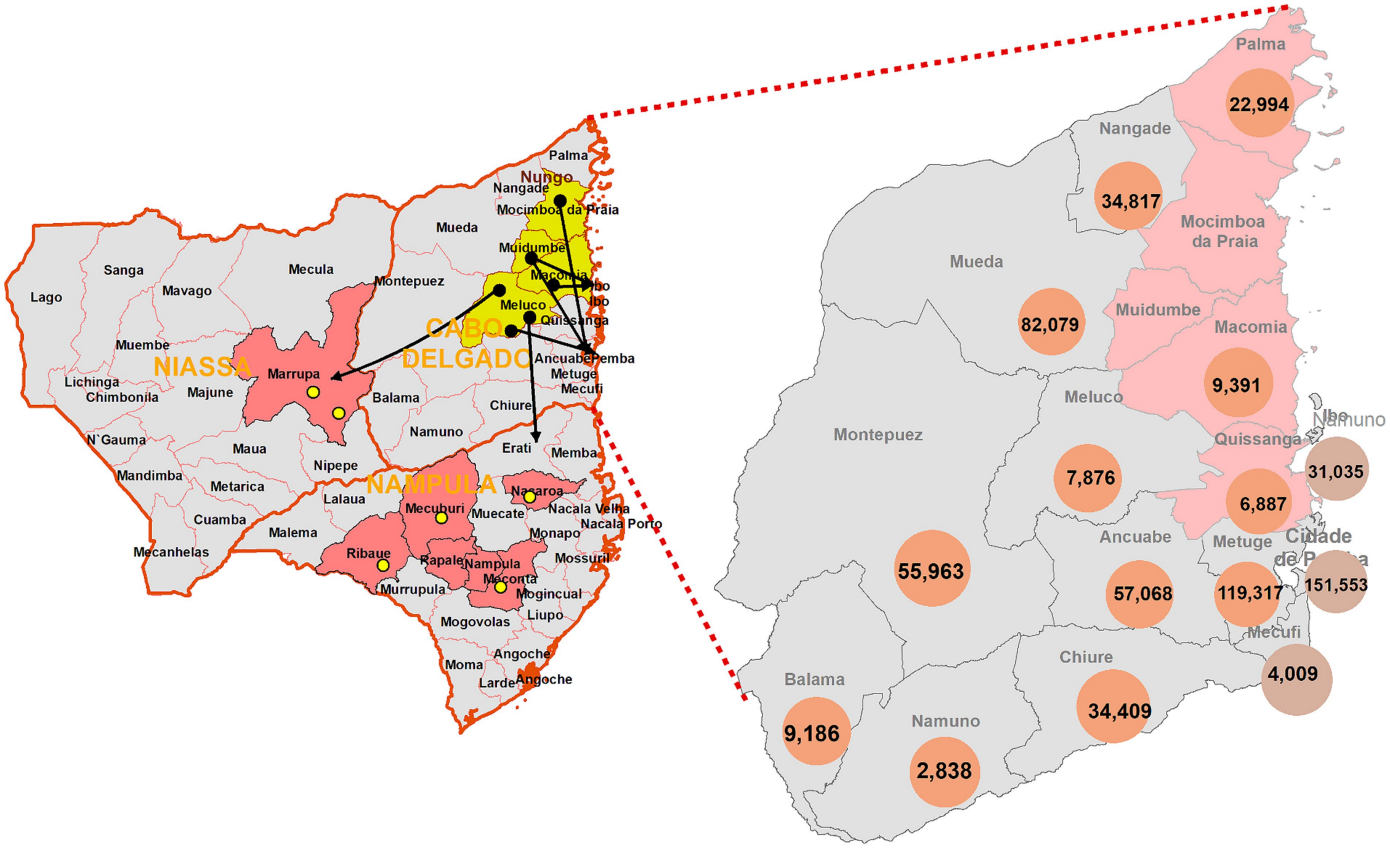
Distribution of internally displaced people in the districts of Cabo Delgado province in March 2021. Source: ADIN, 2020; IOM, 2020, 2021. Available at: https://unhabitat.org/sites/default/files/2021/05/un-habitat_positionpaper_mozambique_pt.pdf.

Metuge district was selected because (1) was considered a “safe” district at the time of the study and close to Pemba City (trip to and from Pemba City on the same day) (32); (2) after the Pemba district, Metuge is the district with the highest number of IDPs resettled (30); (3) has a type 2 health facility close to the IDP resettlement with health professionals trained in the management of mental disorders (Figure 1).

### Study participants and sampling procedure

As the list of all IDPs who are resettled in the selected IDP resettlement was not available, the participants were selected by convenience. There were 7 villages in the reception center (considered as clusters) and the chief of each village was responsible for assisting with recruitment. Based on the time available for the team to be in the field, it was estimated that 50 interviews would be needed per day. So, the aim was to interview around 7–10 participants from each village per day. All houses were contacted by the chief of the village and those who agreed to take part in the study were referred to the project team to be interviewed.

Exposure to war, living in conflict zones, flight, and forced migration may create or increase the risk for broad sequelae of direct and indirect risks for physical and mental health, more so for children and adolescents, even more so for unaccompanied minors separated from their parents; the reason why we included the study participants who were at least 14 years old at the time of data collection. Written informed consent (including the assent term) was obtained from all patients.

### Sample size determination

Based on the International Organization for Migration, Displacement Tracking Matrix Mozambique data, the Metuge district had 119,317 IDPs in March 2021 (30, 33). The sample size was calculated based on a confidence interval of 95% and a margin of error (E) of 5%; without general information on the population distributed in IDP camps, or results from the prevalence of PTSD, depression, or anxiety in previous studies, we considered a prevalence of 50%. Considering a 10% non-response rate and a design effect = 1.5 the minimum sample size was equal to 634.

### Data collection procedures

From January 2023 to April 2023, three trained data collectors, supervised by the first author (NM), administered a structured questionnaire The face-to-face interview took place 1 person at a time for a maximum of 30 min, to ensure privacy. The respondents were given no monetary or food-item incentives. The questions were read aloud to the respondents 1 question at a time during the interview, and the respondents were asked which of the scale choices was acceptable. The coinvestigators reviewed the data collection sheets for completeness, accuracy, and internal consistency, which the principal investigator confirmed.

The interviews were conducted using tablets and included questions of sociodemographic and displacement characteristics, and screening measures for psychiatric disorders (PTSD, depression, and generalized anxiety).

The first section of the questionnaire assessed sociodemographic characteristics, including age (in years), sex (male or female), marital status [status (never married, widowed or divorced, and married), educational level (none school, primary school, and secondary school), religion (Muslim, Catholic, Protestant)], and provenance as well as psychosocial variables such as chronic medical illness (yes or no), whether family members or friends were killed during the armed conflict (yes or no), frequency of being in an armed conflict (1, 2 or 3 and more), last time were exposed to armed conflict (<12 weeks, 12 weeks to 1 year, and >1 year), missing family member (yes or no) were also recorded. Before administering the questionnaire, each respondent listened to the explanations about the aim of the study and terms of participation. The questionnaire was developed by the authors administered in Portuguese and translated into the local language (dialect) whenever necessary.

The outcome measures were PTSD, depression, and anxiety; these were measured using adapted and validated tools for low-and middle-income countries (LMIC) (34–36) including Mozambique (37–39). The primary care PTSD Screen for DSM-5 (PC-PTSD-5) was used to determine the presence of posttraumatic stress symptoms over the last month (40). It is a 5-item screen designed to identify individuals with probable PTSD in primary care settings in high-income countries (41) and it has been validated for use in LMIC adolescents living with HIV infection in South Africa (36) and Mozambique (37). Each of the five items was rated on a binary scale (0 = No, 1 = Yes) (40). Available data suggest the PC-PTSD-5 screen should be considered “positive” if the respondent answers “yes” to any 3 or more items in the questions (40). A score of = 4 was used as a cut-off for this study (41).

Depression was measured by the Patient Health Questionnaire (PHQ-9) with a recall period of the previous 2 weeks (42). The PHQ-9 is one of the most commonly used depression screening instruments (42, 43) and has been validated for use in adults in numerous community studies in LMIC, including Tanzania (34), South Africa (36), and Mozambique (38).

The PHQ-9 is a multipurpose instrument for screening, diagnosing, monitoring, and measuring the severity of depression. Total scores of 5, 10, 15, and 20 represent cut points for mild, moderate, moderately severe, and severe depression, respectively (42). The following cut-offs correlate with level of depression severity: score 0–4: none or minimal depression; score 5–9: mild depression; score 10–14: moderate depression; score 15–19: moderately severe depression; and score 20–27: severe depression (42). The cut-off score of depression screening was 10 or more in this study according to the results of a tool validation study carried out in Mozambique (37, 38).

Anxiety symptoms were assessed by the GAD-7 scale. The GAD-7 is a commonly used instrument to screen for anxiety in high-income countries (44, 45) that has been validated for use in LMIC adults (36, 42) and adolescents from Mozambique (37, 39). GAD-7 total score for the seven items ranges from 0 to 21 with a recall period of the previous 2 weeks (44). When screening for anxiety disorders, a score of 8 or greater represents a reasonable cut-point for identifying probable cases of generalized anxiety disorder (sensitivity of 92% and specificity of 76%) (44, 45). The following cut-offs correlate with the level of anxiety severity: score 0–4: minimal anxiety; score 5–9: mild anxiety; score 10–14: moderate anxiety and score 15–21: severe anxiety (44). The cut-off score of =10 or more was used as the cut-off for anxiety symptoms in this study (36, 37).

### Data analysis

Descriptive statistics, such as percentages, average, median, and standard deviation were used to characterize variables depending on their distribution. We considered three main outcomes (dependent variables): PTSD, anxiety, and depression. The Chi-square or Fisher test was used to assess associations between the main outcomes and the independent variables. To estimate the determinants of PSTD, we calculated crude odds ratio (COR) and adjusted odds ratio (AOR) using logistic regression models.

Variables in the bivariate logistic analysis with a *p* < 0.2 were included in the multivariable model. We take *p* < 0.2 as the rule to include variables in the multivariable model as it is the most common rule available in the literature; as such our study will be comparable with others (46, 47).

We considered a 5% significance level. Statistical analysis was conducted using SPSS software version 28.0 (SPSS Inc., Chicago, Illinois, United States).

## Results

### Sociodemographic characteristics of study respondents

The study included 748 participants of the 750 invited. Of the 748 Metuge IDPs who participated, 487 (65%) were women. The median age of the respondents was 32 (IQ 23–49) years old, with an age range of 14–91 years. More than half of the participants (41.2–55%) were aged between 21 and 44 years old and 521 (69.7%) of the total respondents were married or living with a partner. In terms of occupation, 585 participants (78.2%) were unemployed, 16 were employed (2.1%) and 147 (19.7%) were students.

In addition, 656 of 748 respondents (88%) had a low level of education (35.2–47.1% had a primary school, and 30.4–40.6% did not go to school and could not read or write).

According to the place of provenance, 721 (96.4%) of the IDP participants departed from Quissanga district. In terms of religion, 514 (69%) were Muslim (Table 1).

**Table 1:** Sociodemographic characteristics of study participants from armed conflict area of Metuge district, Cabo Delgado (*n* = 748).

Characteristics	Category	Frequency	%
Age (years)	14–20	120	16.0%
	21–44	410	55.8%
	45–64	151	20.2%
	65+	67	9.0%
Sex	Female	487	65.1%
	Male	261	34.9%
Marital status	Single	179	23.9%
	Married	521	69.7%
	Divorced	19	2.5%
	Widowed	29	3.9%
Level of education	None	304	40.6%
	Primary School	352	47.1%
	Secondary School	92	12.3%
Occupation	Employed	16	2.1%
	Unemployed	585	78.2%
	Student	147	19.7%
Religion	Muslim	514	68.7%
	Protestant	146	19.5%
	Catholics	42	5.6%
	Others^**^	46	6.1%
Provenance	Quissanga	721	96.4%
	Metuge	16	2.1%
	Others^***^	11	1.5%

Others^*^: Orthodox; Others^**^: Macomia, Meluco, and Mocimboa da Praia. Chronic disease^****^ hypertension, mental disease, HIV, diabetes, and asthma. Vulnerable^*****^ physical deficient, pregnant, elderly, and orphan.

#### Potential risk factors for PSTD, depression, and anxiety development

Overall, 114 (15.2%) participants were vulnerable persons (58.8% older adults, 19.2% orphans, 17.5% pregnant, and 4.4% physically deficient). Forty (5.3%) reported the presence of chronic medical illness.

Among the 35 (52.2%) participants with a history of previous mental disorders (anxiety, depression, and epilepsy), 13 (19.4%) reported also having hypertension, 3 (4.5%) HIV infection, 2 (3.0%) diabetes or 2 (3.0%) asthma as self-reported comorbidity.

Regarding trauma exposure, participants reported experiencing a mean of 2.46 trauma events. Approximately 50% (371/748) of IDPs enrolled had been in an attack situation for the third time or more.

About 48% (359/748) of participants lost their family members and 28% (211/748) have witnessed the death of a family member in this war-related event. In terms of the last time that the IDPs were exposed to an attack situation, 65% (484/748) had been in this situation more than 1 year ago (Table 2).

**Table 2:** Distribution of mental disorders among participants from armed conflict area of Cabo Delgado.

Variables	Category	Total (*n* = 748)	PTSD (*N*, %)	Depression (*N*, %)	Anxiety (*N*, %)
Age (years)	14–20	120 (16.0%)	81 (14.6%)	46 (9.6%)	24 (8.0%)
	21–44	410 (55.8%)	313 (56.3%)	270 (56.6%)	165 (55.2%)
	45–64	151 (20.2%)	116 (20.9%)	107 (22.4%)	76 (25.4%)
	65+	67 (9.0%)	46 (8.3%)	54 (11.3%)	34 (11.4%)
Sex	Female	487 (65.1%)	404 (72.7%)	353 (74.0)	220 (73.6%)
	Male	261 (34.9%)	152 (27.3%)	125 (26.0)	79 (26.4%)
Marital status	Single	179 (23.9%)	133 (23.9%)	110 (23.1%)	71 (23.7%)
	Married	521 (69.7%)	392 (70.5%)	334 (70.0)	212 (70.9%)
	Divorced	19 (2.5%)	11 (2.0%)	11 (2.3%)	1 (0.3%)
	Widowed	29 (3.9%)	20 (3.6%)	22 (4.6%)	15 (5.0%)
Level of education	None	304 (40.6%)	252 (45.3%)	230 (48.2%)	139 (46.5%)
	Primary School	352 (47.1%)	246 (44.2%)	195 (40.9%)	125 (41.8%)
	Secondary School	92 (12.3%)	58 (10.4%)	52 (10.9)	35 (11.7%)
Occupation	Employed	16 (2.1%)	8 (1.4%)	4 (0.8%)	5 (1.7%)
	Unemployed	585 (78.2%)	443 (79.7%)	406 (85.1%)	251 (83.9%)
	Student	147 (19.7%)	105 (18.9%)	67 (14.0%)	43 (14.4%)
Religion	Muslim	514 (68.7%)	376 (67.6%)	340 (71.3%)	202 (67.6%)
	Protestant	146 (19.5%)	108 (19.4%)	91 (19.1%)	66 (22.1%)
	Catholic	42 (5.6%)	35 (6.3%)	22 (4.6%)	15 (5.0%)
	Others^*^	46 (6.1%)	37 (6.7%)	14 (2.9%)	16 (5.4%)
Provenance	Quissanga	721 (96.4%)	541 (97.3%)	464 (97.3%)	296 (99.0%)
	Metuge	16 (2.1%)	10 (1.8%)	8 (1.7%)	2 (0.7%)
	Others^**^	11 (1.5%)	5 (0.9%)	5 (1.0%)	1 (0.3%)
Having chronic	No	681 (91.0%)	508 (91.4%)	430 (90.1%)	271 (90.6%)
medical illness^***^	Yes	**67 (9.0%)**	48 (8.6%)	47 (9.9%)	28 (9.4%)
Vulnerable	No	634 (84.8%)	470 (84.5%)	385 (80.7%)	237 (79.3%)
person^****^	Yes	114 (15.2%)	86 (15.5%)	92 (19.3%)	62 (20.7%)
Exposure to cumulative	1–2	377 (50.4%)	292 (52.5%)	239 (50.1%)	130 (43.5%)
trauma events	3+	371 (49.6%)	264 (47.5%)	238 (49.9%)	169 (56.5%)
Last time were	<12 weeks	260 (34.8%)	215 (38.7%)	211 (44.2%)	164 (54.8%)
Exposed	> 12 weeks – 1 years	4 (0.5%)	3 (0.5%)	1 (0.2%)	0 (0.0%)
to attack situation	> 1 year	484 (64.7%)	338 (60.8%)	265 (55.6%)	135 (45.2%)
Missing family	No	389 (52.0%)	295 (53.1%)	251 (52.6%)	157 (52.5%)
Member	Yes	359 (48.0%)	261 (46.9%)	226 (47.4%)	142 (47.5%)
Witnessed the death	No	537 (71.8%)	421 (75.7%)	361 (75.7%)	233 (77.9%)
of a family member	Yes	211 (28.2%)	135 (24.3%)	116 (24.3%)	66 (22.1%)

PTSD, post-traumatic stress disorder; Others^*^: Orthodox; Others^**^: Macomia, Meluco, and Mocimboa da Praia. Chronic Disease^***^ Hypertension, mental disease, HIV, Diabetes, and asthma. Vulnerable^****^ Physical deficient, pregnant, older adult, and orphan.

The prevalence of PTSSs was 56.3% (313 of 556) for IDPs aged 21–44 years and 14.6% (81 of 556) for those younger than 21 years.

Among married participants, approximately 70% had PTSSs, depression, and anxiety, while among the never-married (single) respondents (23%).

We found that 252 of the 556 participants who did not have formal education (45.3%) had PTSSs.

Employed IDP participants had a lower prevalence of PTSSs, depression, and anxiety (1–2%) than unemployed IDP participants (80–85%).

#### Prevalence/frequency of PSTD, depression and anxiety

Of the 748 Metuge IDPs, 556 (74.3%) had post-traumatic stress symptoms (PTSSs) (CI = 71.04–77.42) (27.3% men; 72.7% women). The prevalence of depression was 64% (477 of 748; CI = 60.21–67.22) (26% men; 74% women), and the prevalence of anxiety was 40% (299 of 748; CI 36.44–43.58) (26.4%; 73.6% women). Post-traumatic stress disorder was found in 90.1% (CI 87.11–92.67) and 88.6% (CI 84.47–91.99) of patients with depression, and anxiety symptoms, respectively. The commonest level of depression symptoms was severe (171; 22.9%) followed by moderate depression (163; 21.8%). Nearly one-third of all respondents (224; 29.9%) had moderate anxiety and 75 (10%) had severe anxiety (Table 3).

**Table 3:** Prevalence of post-traumatic stress disorder, depression, and anxiety among the respondents from armed conflict area of Metuge Cabo Delgado, Mozambique.

Characteristics	Category	Overall (*n* = 748)		PTSD		Depression		Anxiety	
		(*N*; %)	CI 95%	(*N*; %)	CI 95%	(*N*; %)	CI 95%	(*N*; %)	CI 95%
PTSD	No	192 (25.7%)	22.57–28.95	–	–	47 (9.9%)	7.33–12.88	34 (11.4%)	8.00–15.52
	Yes	556 (74.3%)	71.04–77.42	–	–	430 (90.1%)	87.11–92.67	265 (88.6%)	84.47–91.99
Depression symptoms	No	271 (36.2%)	32.77–39.79	126 (22.7%)	19.24–26.37	–	–	24 (8.0%)	5.21–11.71
	Yes	477 (63.8%)	60.21–67.22	430 (77.3%)	73.62–80.75	–	–	275 (92.0%)	88.29–94.79
Level of depression symptoms	None/Minimal	159 (21.3%)	18.37–24.36	52 (9.4%)	7.06–12.08	–	–	6 (2.0%)	0.74–4.31
	Mild	112 (15.0%)	12.49–17.73	74 (13.3.%)	10.59–16.42	–	–	18 (6.0%)	3.60–9.34
	Moderate	163 (21.8%)	18.88–24.92	144 (25.9%)	22.30–29.75	–	–	67 (22.4%)	17.81–27.56
	Moderately severe	143 (19.1%)	16.35–22.12	129 (23.2%)	19.75–26.93	–	–	84 (28.1%)	23.07–33.55
	Severe	171 (22.9%)	19.89–26.04	157 (28.2%)	24.53–32.18	–	–	124 (41.5%)	35.83–47.28
Anxiety Symptoms	No	449 (60.0%)	56.41–63.55	291 (52.3%)	48.09–56.55	202 (42.4%)	37.86–46.92	–	–
	Yes	299 (40.0%)	36.44–43.58	265 (47.7%)	43.44–51.91	275 (57.6%)	53.07–62.13	–	–
Level of anxiety Symptoms	None/Minimal	316 (42.2%)	38.67–45.87	177 (31.8%)	27.97–35.88	103 (21.6%)	17.98–25.56	–	–
	Mild	133 (17.8%)	15.10–20.71	114 (20.5%)	17.22–24.10	99 (20.8%)	17.20–24.67	–	–
	Moderate	224 (29.9%)	26.68–33.37	207 (37.2%)	33.20–41.39	204 (42.8%)	38.27–47.34	–	–
	Severe	75 (10.0%)	7.96–12.40	58 (10.4%)	8.01–13.27	71 (14.9%)	11.81–18.40	–	–

PTSD, post-traumatic stress disorder; CI, confidence interval.

### Proportion of post-traumatic stress among the respondents in war-affected area of Cabo Delgado

Having witnessed the death of a family member, depression, and anxiety symptoms had a statistically higher difference than not having witnessed the death of a family member, without the presence of depression and anxiety symptoms (*p* < 0.001) to develop PTSSs (Table 4).

**Table 4:** Proportion of post-traumatic stress among the respondents from armed conflict area of Metuge, Cabo Delgado, Mozambique.

Characteristics	Category	PTSD = No	PTSD = Yes	*p*-value
Having chronic medical illness	No	173 (25.4%)	508 (74.6%)	0.597
	Yes	19 (28.4%)	48 (71.6%)	
Vulnerable person	No	164 (25.9%)	470 (74.1%)	0.769
	Yes	28 (24.6%)	86 (75.4%)	
Exposure to cumulative trauma events	1–2	85 (22.5%)	292 (77.5%)	0.049^*^
	3 or more	107 (28.8%)	264 (71.2%)	
Last time were exposed to attack situation	<12 weeks	45 (17.3%)	215 (82.7%)	
	>12 weeks – 1 year	1 (25.0%)	3 (75.0%)	<0.001^*^
	>1 year	146 (30.2%)	338 (69.8%)	
Missing family member	No	94 (24.2%)	295 (75.8%)	0.327
	Yes	98 (27.3%)	261 (72.7%)	
Witness the death of family member/friend	No	116 (21.6%)	421 (78.4%)	<0.001^*^
	Yes	76 (36.0%)	135 (64.0%)	
Depression symptoms	No	147 (54.2%)	124 (45.8%)	<0.001^*^
	Yes	47 (9.9%)	430 (90.1%)	
Suicide risk	No	145 (35.2%)	267 (64.8%)	<0.001^*^
	Yes	47 (14.0%)	289 (86.0%)	
Anxiety symptoms	No	158 (35.2%)	291 (64.8%)	<0.001^*^
	Yes	34 (11.4%)	265 (88.6%)	

PTSD, post-traumatic stress disorder; ^*^p < 0.05.

### Post-traumatic stress disorder determinants among the respondents in the war-affected area of Cabo Delgado

The chance of having post-traumatic stress disorder was higher in females, in individuals having a family member or close friend injured or killed, and in those being screened positive for depression and anxiety symptoms.

Females had 2.2 times the odds of developing PTSSs than males (AOR = 2.30, 95% CI 1.50–3.51). The odds of developing PTSD were 4.8 times higher among had been exposed to war between 12 weeks a year compared with had been exposed to war for 11 weeks or less (AOR = 5.14, 95% CI 0.40–65.83). Having depressive symptoms (AOR = 8.27, 95% CI = 4.97–13.74) and anxiety symptoms (AOR = 1.45, 95% CI = 0.84–2.50) and suicide ideation (AOR = 1.54, 95% CI = 0.94–2.51) were significantly associated with post-traumatic stress disorder (Table 5).

**Table 5:** Proportion post-traumatic stress disorder and associated factors among the participants from armed conflict area of Metuge district in Cabo Delgado (*n* = 748).

Variable	Categories	Pos-traumatic stress disorder			
		Yes %	No %	COR (95% CI)	AOR (95% CI)
Age (years)	14–20^*^	67.50	32.50	–	–
	21–44	76.34	23.66	1.55^*^ (0.99–2.42)	1.29 (0.67–2.45)
	45–64	76.82	23.18	1.59^*^ (0.93–2.73)	1.05 (0.47–2.37)
	65+	68.66	31.34	1.05 (0.55–2.00)	0.33^*^ (0.13–0.82)
Sex	Male^*^	58.24	41.76	–	–
	Female	82.96	17.04	3.49^*^ (2.48–4.90)	2.30^*^ (1.50–3.51)
Educational status	None^*^	82.89	17.11	–	–
	Primary School	69.89	30.11	0.48^*^ (0.32–0.69)	0.59^*^ (0.36–0.96)
	Secondary School	63.04	36.96	0.35^*^ (0.21–0.59)	0.38^*^ (0.19–0.77)
Occupation	Employed	50.00	50.00	–	–
	Unemployed	71.43	28.57	3.11^*^ (1.14–8.46)	0.72 (0.22–2.35)
	Student	75.73	24.27	2.5^*^ (0.88–7.09)	1.49 (0.43–5.14)
Exposure to cumulative trauma events	1–2	79.30	20.70	–	–
	3 or more	71.27	28.73	0.64^*^ (0.45–0.92)	0.66^*^ (0.43–1.01)
Last time were exposed to war fighting	<12 weeks	82.69	17.31	–	–
	>12 weeks – 1 year	75.00	25.00	0.62 (0.06–6.17)	5.14^*^ (0.40–65.83)
	>1 year	69.83	30.17	0.48^*^ (0.33–0.70)	1.24 (0.74–2.08)
Witness the death of family member/friend	No^*^	78.40	21.60	–	–
	Yes	63.98	36.02	0.49^*^ (0.34–0.69)	0.61^*^ (0.39–0.93)
Depression symptoms	No^*^	46.49	53.51	–	–
	Yes	90.15	9.85	10.52^*^ (7.16–15.46)	8.27^*^ (4.97–13.74)
Suicide risk	No^*^	64.81	35.19	–	–
	Yes	86.01	13.99	3.34^*^ (2.31–4.83)	1.54^*^ (0.94–2.51)
Anxiety symptoms	No^*^	64.81	35.19	–	–
	Yes	88.63	11.37	4.23^*^ (2.82–6.35)	1.45^*^ (0.84–2.50)

^*^p < 0.2. AOR, adjusted odds ratio; CI, confidence interval; COR, crude odd ratio.

## Discussion

Nearly 120, 000 IDPs have lived in the resettle camps of the Metuge district since October 2017. During the displacement and post-displacement periods, IDPs faced multiple stressors (48). They live in small, overcrowded temporary shelters in the camps, without sufficient food, clean water, or toilets. Their lives are on hold, and their futures are uncertain. Individuals with an experience of abuse/violence were at risk of increasing mental health problems (17). This study provides a detailed view of the symptoms of traumatic distress (PTSD, depression, and anxiety) encountered by 25 de Junho resettle camps in Metuge district, Cabo Delgado province.

This study found a high prevalence of PTSD, depression, and anxiety symptoms in IDPs in Cabo Delgado province. People affected were relatively young, with 71.8% under the age of 44, and predominantly female (65%). The rate of illiteracy was 41% with 80% of respondents unemployed and 96.4% departed from Quissanga district-which is among the most affected by the armed violence affecting the province since October 2017 (30).

Our study participants had lower educational levels (90%) and a 41% illiteracy rate and women are almost twice as likely to be illiterate as men in concordance with the national average (39.9%) (32). The Cabo Delgado province, despite being abundantly rich in mineral and environmental resources, has the highest rate of illiteracy (52.4%; 12.5% above the national average in population aged 15 and above) and multidimensional poverty in the country (32).

Additionally, the study participants had a high unemployment rate (78%) which is in concordance with the last report of the National Statistical Institute in Mozambique (32). The aggregate prevalence of depression, anxiety, and post-traumatic stress was considerably high, with a prevalence rate of 63.8, 40.0 and 74.3%, respectively, when compared with other studies conducted in armed conflict-affected populations (10.8% for depression, 15.3% for PTSD and 21.7% for anxiety) according to a systematic review and meta-analysis (15). Another systematic review (1981–2014) from six countries, five in Africa (18 studies), reported that the most frequent being post-traumatic stress disorder (range 3.1–75.9%), anxiety (range 6.9–75%), and depression (range 8.8–76.5%) (49).

The prevalence of PTSD at 74.3% was in line with the study carried out in Uganda (74%) (50) and lower than a study done in Medellin Colombia (88%) (51) and Iraq (83.4%) (52). Contrarily, the estimated PTSD prevalence of the current study was higher than the studies carried out as 63% in Nigeria (21), 40.8% in northwest and 58.4% in south of Ethiopia (24, 46), 19.3% in Morocco (22), 28% in South Sudan (23), 29.9% in Somalia (53) and 7.7% in Sri-Lanka (54), 46.6% in Bangladesh (55) particularly among women (5, 24, 55, 56).

The possible explanation for the observed difference could be the difference in tools, in which northwest Ethiopia (46), and northeast Ethiopia (56) studies used the post-traumatic stress disorder checklist for DSM (PCL-5), in Sri Lanka and Sweden studies used the Harvard trauma questionnaire (HTQ) (54, 57), and in Somalia and Uganda studies were used the International Neuropsychiatric Interview (MINI) (53, 58).

The high prevalence of PTSSs suggests that a scale-up of mental health care is needed, which could be met by increasing medical workers’ capacity in the Metuge health facilities to diagnose and treat patients with mental disorders. However, the current resources for mental health services are insufficient in the Metuge district, and the number of mental health professionals (2) is too low to cover the entire Metuge population in need.

The prevalence of depression symptoms (63.8%) was higher than the studies carried out in Uganda (58%) (58), in the south (53.3%) (24) and North Ethiopia (39.3%) (46), Sweden (40.2%) (57), Sri-Lanka (22.2%) (54) and Somalia (32.1%) (53). Contrarily, the estimated depression symptoms prevalence of the current study was lower than the study carried out at 89% in Bangladesh (59).

Additionally, the prevalence of anxiety symptoms (40.0%) in the current study was lower than the study done in southwestern Uganda (73%) (58). On the other hand, the findings of the current study were higher than the study done in northeast Ethiopia (33.4%) (56), Sweden (31.8%) (57), Somalia (34.9%) (53), and Sri Lanka (32.6%) (54). A possible theory to explain the high prevalence of PTSD, depression, and anxiety symptoms may be the repeated exposure to war attacks and/or violence (previous and current armed conflict, natural disasters, and COVID-19 pandemic) (60) that these displaced individuals had been exposed to several moments (the last attack in Quissanga district being recorded 6 months before the beginning of the study) (61). Of note, the region has recorded extreme weather events, such as Cyclone Kenneth in April 2019, affecting around 500,000 households that saw their homes partially or destroyed, followed by the torrential rains recorded in December 2019 and January 2020 (62). Furthermore, the COVID-19 pandemic and several outbreaks of cholera caused limited access to essential health services and thousands of additional deaths (63).

PTSD was significantly associated with being female (2.2. times), suicide risk (1.54 times), being screened positive for depression (8.27 times), and generalized anxiety (1.45 times).

Approximately half the respondents in our sample were female. The study findings show that mental health symptoms were more prevalent in female IDPs than in male IDPs. The odds of developing PTSD were 2.3 times higher in females compared to males, in line with the findings of other studies done in the south (24, 64) and north of Ethiopia (46, 56), and the fact that females have a higher risk of developing PTSD due to a lower threshold from exposure to psycho-trauma compared to males (51). In addition, many studies show that women were found to have a higher incidence of mental health disorders after rape or sexual assault (12, 65), the violent loss of a partner, or children, and becoming a single parent or widow (50, 66). Another reason could be that females tend to show a more emotional and ruminative response to stress (67). Contrarily, some studies found that symptoms of traumatic distress were more prevalent among male refugees than female refugees. (12, 55, 65)

Our research also found that IDPs who were unable to read or write had a higher prevalence of mental health symptoms (PTSD, depression, and anxiety) than those who had schooling. This finding is comparable to previous research that showed that poor education level was correlated to higher rates of PTSD (55, 68, 69) and increased risk of developing PTSD, depression, and anxiety (70).

Also, married respondents were more likely to have PTSSs, depression, and anxiety than those who were never married. This finding is comparable to findings in previous research (69).

Participants with depression were 8 times more likely to have PTSD when compared to participants without depression. Among patients with PTSD, depressive disorders, anxiety disorders, and drug misuse are 2 to 4 times more prevalent than among patients without PTSD (71). This is consistent with research conducted in Ethiopia (24, 56), Uganda (58), and Kenya (72). This has been related to participants with depression being more likely to have suffered traumatic experiences compared to participants without depression (67). Moreover, PTSD may increase the risk of suicide attempts (73).

### Limitations

The results we present derive from a single center sample of IDP. The study participants were selected by convenience; and more representation of females may have impacted the results. Due to the cross-sectional nature of the study, we were not able to verify whether the depression, anxiety symptoms, and substance use, preceded or followed PTSD. Despite this, the findings may be helpful to nations with areas devastated by armed conflicts or war.

## Conclusions and recommendations

The high prevalence of self-reported mental health symptoms in this study was found in a displaced working-age population. Being female, having a clinical feature of depression and anxiety, and having antecedents of the death of a family member, were associated with the development of PTSD. These results highlight the need for surveillance and follow-up studies, and training on stress management for their violent memories in the context of humanitarian emergencies in displaced populations, aiming to early diagnose and deliver group or individual psychological support, may reduce the burden of severe mental health symptoms. Likewise, these results highlight the need for more evidence-based research specifically from policymakers and stakeholders at the national and global level to tackle the common mental disorders issues through intervention.

## Data availability statement

The raw data supporting the conclusions of this article will be made available by the authors, without undue reservation.

## Ethics statement

The studies involving humans were approved by National Bioethics Committee for Health of Mozambique with registration IRB00002657. The studies were conducted in accordance with the local legislation and institutional requirements. Written informed consent for participation in this study was provided by the participants’ legal guardians/next of kin.

## Author contributions

NM: Conceptualization, Data curation, Formal analysis, Funding acquisition, Investigation, Methodology, Project administration, Resources, Software, Visualization, Writing – original draft, Writing – review & editing. HI-M: Investigation, Writing – review & editing. FP: Formal analysis, Software, Writing – review & editing. PD: Validation, Writing – review & editing. FM: Conceptualization, Methodology, Writing – review & editing. VC: Validation, Writing – review & editing. AM: Supervision, Validation, Writing – review & editing. MM: Formal analysis, Funding acquisition, Software, Supervision, Validation, Writing – review & editing.
